# Braithwaite’s Retrospect

**Published:** 1860-03

**Authors:** 


BRAITHWAITE'S RETROSPECT.—An epitome of this work, from its commencement to the present time, has been prepared by Dr. Walter S. Wills, and is to be issued in five parts, at one dollar each. The first number has just been published at New York, forming a handsome octavo of upwards of 300 pages. The original work is comprised in forty volumes, the complete set costing no less than thirty dollars. The abridgement, which seems to have been prepared with great care, should be in the library of every young physician in the country.

